# Endovascular Thoracoabdominal Replacement after Total Abdominal Aortic Debranching

**DOI:** 10.1055/s-0038-1641607

**Published:** 2018-07-27

**Authors:** Murat Ugurlucan, Yilmaz Onal, Omer Ali Sayin, Feza Ekiz, Didem Melis Oztas, Murat Basaran, Bulent Acunas, Ufuk Alpagut

**Affiliations:** 1 Department of Cardiovascular Surgery, Istanbul University Istanbul Medical Faculty, Istanbul, Turkey; 2 Department of Radiology, Istanbul University Istanbul Medical Faculty, Istanbul, Turkey; 3 Department of General Surgery, Istanbul University Istanbul Medical Faculty, Istanbul, Turkey

**Keywords:** Marfan syndrome, aneurysm, dissections, endovascular stent graft

## Abstract

Marfan syndrome is an inherited connective tissue disorder affecting mainly eyes and skeletal and cardiovascular systems. Cardiovascular involvement may lead to life-threatening aortic pathologies including aneurysms and/or dissections. In this report, the authors present images of a patient with Marfan syndrome with a history of Bentall-De Bono procedure followed by aortic arch and infrarenal aortoiliac replacements who strongly refused conventional open repair and underwent abdominal debranching followed by thoracoabdominal endovascular stent grafting for the treatment of thoracoabdominal aneurysm.

Marfan syndrome is a worldwide distributed, well-known autosomal dominant inherited connective tissue disorder affecting mainly the eyes and skeletal and cardiovascular systems. Cardiovascular involvement may lead to life-threatening aortic pathologies including aneurysms and/or dissections. 1

A 27-year-old male Marfan syndrome patient with a history of Bentall-De Bono, aortic arch, and infrarenal abdominal aortic replacements was referred with the diagnosis of giant thoracoabdominal dissecting aneurysm. Treatment options, especially the benefits of conventional thoracoabdominal replacement in this young patient with the particular connective tissue disorder, were explained in detail and discussed with the patient; however, he strongly refused the standard therapy. The alternative, that is, total abdominal debranching followed by endoluminal stenting was the other option and he wanted to receive this therapy despite similar risks of paraplegia with open surgery.

He underwent abdominal aortic debranching (from right iliac artery to the right renal artery and right iliac artery to left renal, superior mesenteric, and celiac artery) followed by endovascular thoracoabdominal stent graft implantation between the aortic arch and infrarenal abdominal aortic grafts, for the treatment of the pathology ( Fig. 1 , Video 1 ). There was no significant blood loss and no blood or blood product transfusion was required.

**Fig. 1:**
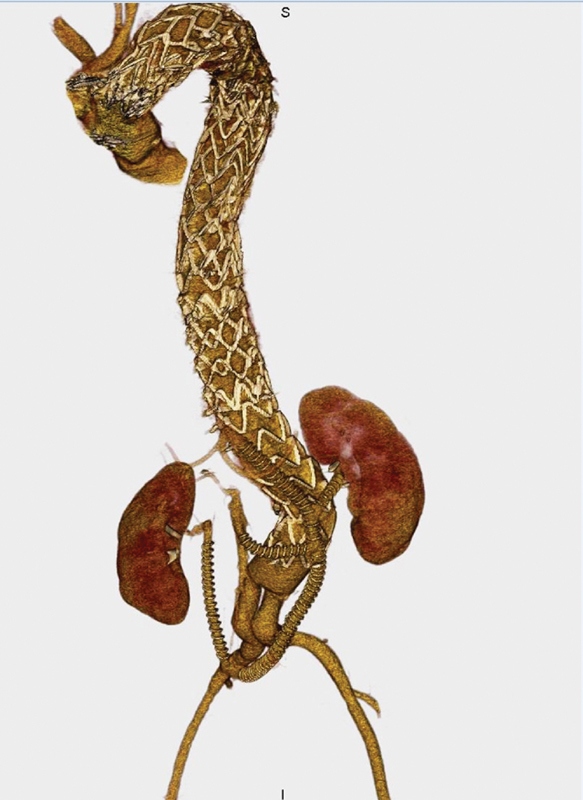
Aortic debranching (right iliac artery to right renal artery and right iliac artery to celiac, superior mesenteric and left renal artery) followed by thoracoabdominal endovascular stent grafting.

**Video 1**

Cine of the postoperative control computed tomography angiography. Online content is viewable at: https://www.thieme-connect.com/products/ejournals/html/10.1055/s-0038-1641607 .

Conventional thoracoabdominal replacement carries serious mortality and morbidity risks including visceral organ dysfunction, paraplegia, and consequences of cardiopulmonary bypass. 2 Although our treatment strategy offered a less invasive alternative to surgical thoracoabdominal aortic replacement, the current article presents only the images of our technique and we did not aim to propose visceral debranching followed by endovascular thoracoabdominal stent graft implantation as a new and safe alternative to open surgery. This is certainly a very controversial topic of treating very young patients with known connective tissue disorders in an endovascular fashion. Literature includes case reports and very small series. 3 However, usually these patients are older and have prohibitive risk for an open procedure. In a young man of 27 years, this may be accepted as borderline. There is risk of paraplegia no matter which approach, that is, endovascular or open, is chosen for the entire aortic replacement and most endovascular series still assure the use of all adjuncts to ameliorate this risk. 4 A hybrid approach is not necessarily a lower risk surgery than a thoracoabdominal approach as demonstrated in experiences of others on hybrid techniques. 5 We believe debranching followed by endoluminal stent graft implantation is a possible treatment option for thoracoabdominal aortic aneurysms; however, it should be considered in selected patients with certain comorbidity factors as well as in whom open techniques are strongly rejected.

## Notes

Discussion—Questions and Answers

1. How high would you estimate the risk of paraplegia to be for your endovascular Marfanoid?
  Interestingly, although we completely covered the whole thoracoabdominal aorta with stent grafts, paraplegia did not occur. It should not mean that if the procedure is performed again, paraplegia will never occur. Definitely paraplegia is a certain risk; however, it did not occur in our patient.
2. How is the spinal cord receiving its blood supply now?
  Although the whole aorta is covered, we assume the blood supply to the spinal cord should be through collaterals from the subclavian arteries or the internal iliac arteries.
3. Are you concerned about radial force on the Marfanoid aortic tissue?
  The patient had Marfan syndrome and experienced multiple aortic problems. When he was admitted with thoracoabdominal aneurysm, we strongly recommended conventional thoracoabdominal replacement, as the benefits of this method on Marfanoid tissue have been shown. However, he refused any major surgery including cardiopulmonary bypass as he had experienced cerebral complications of total circulatory arrest (unconsciousness for 4 days after surgery, loss of orientation for a few weeks …etc.) during his aortic arch replacement. He was then treated upon his consent and acceptance of frequent clinic follow-up. Definitely we are concerned about the radial force on the Marfanoid aortic tissue and patient is advised to immediately reach the hospital if he feels any kind of back pain. In addition, ultrasonography was performed with 3-month intervals and after every 6 months he received computed tomography (CT) examinations. The diseased aortic tissue is still in situ being constantly pushed out with the radial force of the stent grafts. However, we are not sure whether we are placing added strain to the aortic tissue and potentially cause a rupture. Since the aortic flow is directed into the grafts without detectable leak, we assume native aorta is under lower stress. Although the phenomenon has to some degree also been shown by case reports where endografts were placed to bridge therapy and aortic enlargement was noticed on routine follow-up imaging on this patient population, so far our follow-up data did not indicate endoleak, aortic enlargement, or rupture.
4. How durable do you expect your repair to be in this young patient?
  Now that there is a stent graft covering the thoracoabdominal dissecting segment of the aorta. Several questions may arise: (1) the need for yearly CT angiography follow-up for the lifetime of this patient, which can place his kidneys at risk of certain damage; (2) since the stenting was performed in the setting of dissecting disease, endoleaks may be encountered, specifically from his intercostal arteries, which did not show perfusion in the false lumen of the dissecting segment in the follow-up control films. The durability of the particular repair in this Marfanoid young patient should have three major aspects including the lifetime paraplegia risk, the patency of the debranching grafts as well as anastomotic aneurysms, and the behavior of the covered aortic tissue. Although he has been followed up event free, he is in lifelong follow-up program and needs to be documented if a complication occurs.
